# Global Globin Network and adopting genomic variant database requirements for thalassemia

**DOI:** 10.1093/database/baae080

**Published:** 2024-09-04

**Authors:** Hashim Halim-Fikri, Ninie Nadia Zulkipli, Hafiza Alauddin, Celeste Bento, Carsten W Lederer, Petros Kountouris, Marina Kleanthous, Yetti Hernaningsih, Meow-Keong Thong, Muhammad Hamdi Mahmood, Norafiza Mohd Yasin, Ezalia Esa, Jacques Elion, Domenico Coviello, Raja-Zahratul-Azma Raja-Sabudin, Ghada El-Kamah, John Burn, Narazah Mohd Yusoff, Raj Ramesar, Bin Alwi Zilfalil

**Affiliations:** School of Medical Sciences, Universiti Sains Malaysia, Health Campus, Jalan Raja Perempuan Zainab II, Kubang Kerian, Kelantan 16150, Malaysia; School of Medical Sciences, Universiti Sains Malaysia, Health Campus, Jalan Raja Perempuan Zainab II, Kubang Kerian, Kelantan 16150, Malaysia; School of Biomedicine, Faculty of Health Sciences, Universiti Sultan Zainal Abidin, Gong Badak Campus, Kuala Nerus, Terengganu 21300, Malaysia; Department of Pathology, Faculty of Medicine, Universiti Kebangsaan Malaysia, Jalan Yaacob Latif, Bandar Tun Razak, Cheras, Kuala Lumpur 56000, Malaysia; Department of Hematology, Hospital Pediátrico de Coimbra, Avenida Afonso Romão, Coimbra 3000-602, Portugal; Molecular Genetics Thalassaemia Department, The Cyprus Institute of Neurology & Genetics, 6 Iroon Avenue, Ayios Dometios, Nicosia 2371, Cyprus; Molecular Genetics Thalassaemia Department, The Cyprus Institute of Neurology & Genetics, 6 Iroon Avenue, Ayios Dometios, Nicosia 2371, Cyprus; Molecular Genetics Thalassaemia Department, The Cyprus Institute of Neurology & Genetics, 6 Iroon Avenue, Ayios Dometios, Nicosia 2371, Cyprus; Department of Clinical Pathology, Faculty of Medicine Universitas Airlangga, Dr. Soetomo Academic General Hospital, Surabaya, East Java 60132, Indonesia; Department of Paediatrics, Faculty of Medicine, Universiti Malaya, Lembah Pantai, Kuala Lumpur 50603, Malaysia; Department of Basic Medical Sciences, Faculty of Medicine and Health Sciences, Universiti Malaysia Sarawak, Kota Samarahan, Sarawak 94300, Malaysia; Haematology Unit, Cancer Research Centre, Institute for Medical Research, National Institutes of Health, No. 1, Jalan Setia Murni U13/52, Seksyen U13, Bandar Setia Alam, Shah Alam, Selangor Darul Ehsan 40170, Malaysia; Haematology Unit, Cancer Research Centre, Institute for Medical Research, National Institutes of Health, No. 1, Jalan Setia Murni U13/52, Seksyen U13, Bandar Setia Alam, Shah Alam, Selangor Darul Ehsan 40170, Malaysia; Medical School, Université Paris Diderot, Paris 75018, France; Laboratorio di Genetica Umana, IRCCS Istituto Giannina Gaslini, Largo Gerolamo Gaslini 5, Genova 16147, Italy; Department of Pathology, Faculty of Medicine, Universiti Kebangsaan Malaysia, Jalan Yaacob Latif, Bandar Tun Razak, Cheras, Kuala Lumpur 56000, Malaysia; Clinical Genetics Department, Human Genetics and Genome Research Institute, National Research Centre, Cairo 12622, Egypt; Translational and Clinical Research Institute, Newcastle University, International Centre for Life, Times Square, Newcastle upon Tyne NE1 3BZ, United Kingdom; Molecular Genetics Section, Clinical Diagnostic Laboratory, Advanced Medical and Dental Institute, Universiti Sains Malaysia, Bertam, Kepala Batas, Pulau Pinang 13200, Malaysia; Division of Human Genetics, Institute of Infectious Disease and Molecular Medicine, Faculty of Health Sciences, University of Cape Town, Observatory 7925, South Africa; Human Genome Centre, School of Medical Sciences, Universiti Sains Malaysia, Health Campus, Jalan Raja Perempuan Zainab II, Kubang Kerian, Kelantan 16150, Malaysia

## Abstract

Thalassemia is one of the most prevalent monogenic disorders in low- and middle-income countries (LMICs). There are an estimated 270 million carriers of hemoglobinopathies (abnormal hemoglobins and/or thalassemia) worldwide, necessitating global methods and solutions for effective and optimal therapy. LMICs are disproportionately impacted by thalassemia, and due to disparities in genomics awareness and diagnostic resources, certain LMICs lag behind high-income countries (HICs). This spurred the establishment of the Global Globin Network (GGN) in 2015 at UNESCO, Paris, as a project-wide endeavor within the Human Variome Project (HVP). Primarily aimed at enhancing thalassemia clinical services, research, and genomic diagnostic capabilities with a focus on LMIC needs, GGN aims to foster data collection in a shared database by all affected nations, thus improving data sharing and thalassemia management. In this paper, we propose a minimum requirement for establishing a genomic database in thalassemia based on the HVP database guidelines. We suggest using an existing platform recommended by HVP, the Leiden Open Variation Database (LOVD) (https://www.lovd.nl/). Adoption of our proposed criteria will assist in improving or supplementing the existing databases, allowing for better-quality services for individuals with thalassemia.

**Database URL**: https://www.lovd.nl/

## Introduction

### Global epidemiology of hemoglobinopathies including thalassemia

There are approximately 270 million carriers of abnormal hemoglobins and thalassemia worldwide, with 80 million of them carrying β-thalassemia [1]. However, these numbers do not fully represent the overall impact of thalassemia, which not only affects individuals with symptoms but also their families, leading to socio-economic challenges.

The resistance of hemoglobinopathy carriers to malaria has caused an increase in alleles in historic malaria regions, including large parts of Africa for sickle cell disease (SCD) and for thalassemia a geographic belt stretching from sub-Saharan Africa and the Mediterranean into the Middle East and South-East Asia [2]. In LMICs located in this thalassemia belt, the pressure of malaria has resulted in thalassemia becoming the most prevalent genetic disease. While some LMICs have made progress in reducing thalassemia cases, the majority are still striving to control the disease and lessen its impact on health [3].

### Thalassemia and its overriding challenge for low- and middle-income countries

Hemoglobinopathies are disorders that affect the expression of the α-globin (HBA) or β-globin (HBB) components of hemoglobin. SCD is the most common type caused by a variant of HBB that promotes the formation of hemoglobin aggregates at low oxygen pressure. β-thalassemia, on the other hand, is characterized by low production of β-globin. These disorders have a well-understood pathophysiology but present a growing global health challenge due to migration and new genotypes. SCD and thalassemia severity are determined by a combination of hundreds of different globin variants and genetic modifiers, which makes phenotype predictions difficult, even when HBA and HBB genotypes are known.

Individuals with thalassemia may have severe anemia, requiring costly blood transfusion and iron chelation for survival. Without treatment, severe cases can lead to death in the first decade of life. Adequate blood transfusion and monitoring, along with proper iron-chelation therapy, are crucial for good quality of life with minimal complications. However, providing safe blood and coordinating national-level transfusion and therapy is challenging in LMICs, where the majority of transfusion-dependent thalassemia (TDT) patients reside [4] and where approximately 80% of newborns with hemoglobinopathies are born [5, 6].

Thalassemia management involves an accurate diagnosis and comprehensive clinical care, including emotional, social, and financial support. Diagnosis requires a comprehensive clinical history, hematology and hemoglobin analyses, and molecular tests. However, access to diagnostic tests is sometimes limited, particularly in LMICs. What is more, with growing populations and improving healthcare facilities, the disease burden is expected to increase due tooverwhelming transfusion and budget capabilities for adequate thalassemia patient care, unless effective prevention programs are implemented.

### Thalassemia management and control

HICs have successfully implemented national hemoglobinopathy control programs, but LMICs face financial, political, and infrastructural challenges in doing so [7]. Some LMICs, such as Turkey and Malaysia, have reported a reduction in thalassemia cases [8–10], while others, such as Pakistan and Iraq, continue to struggle despite implementing prevention programs. A systematic categorization of LMICs according to established thalassemia supports infrastructures [7] and the comparison of effective with ineffective control efforts may guide future programs. The failure of prevention programs in some countries is due to factors such as lack of training, limited awareness, poor coordination, and inexperienced support organizations [11].

Hemoglobinopathies are expected to become more prevalent due to poor management and migration of affected populations to non-endemic regions. This will result in an increasing health burden in HICs where hemoglobinopathies are frequently underdiagnosed, and health systems are often unresponsive. Poor coordination of international efforts and a lack of supportive infrastructures will exacerbate this issue, which may impose a strain on the health systems of both LMICs and HICs, increasing the global health burden of hemoglobinopathies [6].

Diagnostic laboratories in industrialized countries are categorized into tiers 1–4 based on the level of specialization, whereas LMICs aim to achieve an integrated network of tiered labs that provide broadly accessible, sustainable, and high-quality pathology services. Ethiopia’s model has found endorsement in the Freetown Declaration of October 2015 as a basis for effective healthcare [12]. Bilateral international support and geographical proximity can also improve healthcare in LMICs, as shown by Thailand’s support for Laos PDR and the establishment of thalassemia diagnosis in Laotian peoples [13].

### Poor records on cases and trends

Importantly, the existence of a database of core information can guide prevention and management strategies for endemic genetic diseases. Countries on the thalassemia belt, including Bangladesh, Sri Lanka, Brunei, Laos, and Myanmar, have varying levels of success in disease control and report a lack of vital data on thalassemia carriers, affected births, and disease prevention initiatives [7, 14, 15]. In particular, little is known about hemoglobinopathy disease prevalence, clinical course, mortality, co-morbidities, and treatment outcomes in Bangladesh [15]. Sri Lanka has implemented a screening program to identify thalassemia carriers and increase awareness, but there is no evidence of its impact on reducing the number of births affected by thalassemia. The absence of a national registry or record of patient numbers and distributions may contribute to such lack of observable impact, hindering prevention efforts and effective resource management [14].

## Tackling hemoglobinopathies

### Economic burden of thalassemia

Economic burden-of-disease (BOD) or cost-of-illness (COI) studies help finance and plan a country’s health program [4, 16]. A COI study catalogues, quantifies, and calculates a problem’s combined costs to assess a disease’s economic impact on society [17]. Multiple countries have reported the economic burden of thalassemia. The anticipated annual cost for a thalassemia patient in Sri Lanka, India, Thailand, and Iran was reported as USD 2601 [18], USD 1135 [19], USD 950 [20, 21], and USD 833 [20], respectively, which is comparatively low compared to that for some other LMICs. However, in Malaysia, the estimated annual cost of α- and β-halassemia from treatment amounts to USD 10 499 [4]. Malaysia follows international thalassemia treatment guidelines similar to those in the UK and the USA[4]. Since most thalassemia patients receive regular blood transfusions and free iron-chelation therapy, Azman and colleagues (2016) found that Malaysia has a higher economic burden than other Southeast Asia (SEA) countries. By contrast, cost-effective screening and prevention programs have reduced thalassemia births in Cyprus, Israel, Sardinia, and Singapore [22–24] and make exceptional levels of care affordable. With their cost reduction compared to life-long therapy, comprehensive national screening programs are thus essential for economic growth [4].

### The burden of thalassemia and hemoglobinopathy in India

β-thalassemia is the most prevalent hemoglobinopathy in India, which globally occupies a significant position in the thalassemia belt. India has the highest number of patients with thalassemia major, with roughly 150 000 cases documented internationally. At between 10 000 and 15 000 new cases reported each year, India accounts for 25% of the worldwide burden of β-thalassemia. Based on data from the 2011 Census of India, the mean prevalence of the β-thalassemia trait among the 1.21 billion inhabitants of India is estimated to be around 42 million carriers (3–4%) [25]. Population screening has revealed a higher frequency of carrier status in several communities, including Lohanas, Sindhis, Gujaratis, Punjabis, Kolis, Mahars, Bengalis, and tribal groups of Gujarat and Odisha. Even so, the exact number of β-thalassemia traits remains inconclusive. The most affected regions are the western and eastern parts of India. Tribal populations account for higher prevalence of β-thalassemia major, β-thalassemia trait, and other hemoglobinopathies than non-tribal populations. Furthermore, low socioeconomic position is associated with a greater carrier rate of β-thalassemia [26]. Colah *et al*. report that managing β-thalassemia patients in India poses a significant national health burden [25]. The annual treatment cost for a β-thalassemia major patient is estimated to be between US$629.00 and US$2300.00, with only a few cases fully handled [27]. For example, the cost of transfusing and chelating a child weighing 30 kg was projected to be USD 2985 a year [28]. As the child grows older, a comprehensive approach to therapy is required, which immediately increases the management cost [29]. Several risk factors contributing to the increase of β-thalassemia patients in India have been identified, such as consanguineous marriage (which accounts for 14% of cousin marriages) [30], insufficient public awareness [31], inadequate motivation, high cost of the test, and a dearth of screening infrastructure in the vicinity [32].

India not only faces a large economic burden on the management of thalassemia, but it also deals with SCD [33]. Approximately 120 000 individuals from India were afflicted with SCD in the year 2016 [34]. As per the National Guidelines for Hemoglobinopathies (NHM 2016), the annual expenses for treating SCD, which includes penicillin prophylaxis, are around USD 45 per child. However, this cost does not include the expenditures associated with hospitalization for transfusions and the overall management of SCD [29]. India can reduce the national health burden through the implementation of a number of preventive strategies, such as the development of low-cost molecular diagnostic tests, massive public awareness and education campaigns at all stages of life, emphasizing the significance of prenatal screening and hemoglobinopathies, and the provision of low-cost molecular diagnosis, particularly in rural areas [31], and implementing limitations on the delivery of affected infants at the state and national levels [26].

### Progress in molecular techniques in thalassemia diagnosis

**Figure 1.**
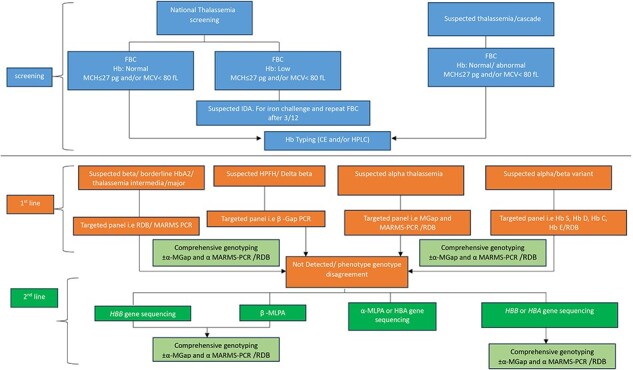
Current workflow for thalassemia screening and diagnosis using conventional PCR (multicenter approach).

Molecular analysis is needed to predict disease severity, from severe, transfusion-dependent thalassemia cases to mild cases that have never been transfused [35] but may have worse survival rates [36], and useful to make accurate diagnoses in cases with complex genetic interactions. There is a plethora of existing molecular techniques that have been used to detect thalassemia, such as reverse dot blot analysis [37, 38], gap-polymerase chain reaction (Gap-PCR) [37, 39], single-tube multiplex amplification refractory mutation system PCR (ARMS-PCR) [40, 41], multiplex ligation-dependent probe amplification (MLPA) [35, 42–44], loop-mediated isothermal amplification (LAMP) [45, 46], Sanger sequencing [47, 48], denaturing gradient gel electrophoresis (DGGE) [49–51], single-strand conformation polymorphism (SSCP) [52, 53], allele-specific PCR (AS-PCR) [54], SNaPshot minisequencing [55], and restriction fragment length polymorphism (RFLP) [49, 56]. As shown in Fig. 1, the legacy techniques based on conventional PCR are laborious, involve multistep analyses and diagnosis, and the decision to choose the panel is based on presumptive diagnosis from complete blood count (CBC) and Hb typing, and thus may lead to misdiagnosis.

**Figure 2.**
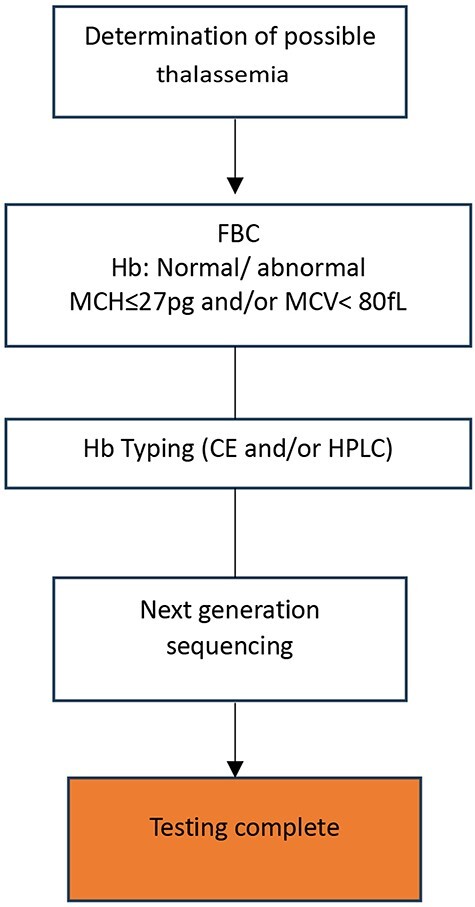
Proposed workflow for thalassemia screening and diagnosis using next-generation sequencing (NGS).

Each of these methods has drawbacks, such as long run or hands-on times [57], low resolution, the need for design optimization and/or expensive mutation-specific probes, or the inability to detect point mutations, small mutations, and deletions [58]. Massively parallel sequencing (MPS, aka next-generation sequencing, NGS) has become widely available and integrated by many laboratories in routine genetic disease diagnostics, including thalassemia, to overcome the limitations of existing techniques. Through whole-genome sequencing, exome sequencing, or targeted gene-panel analysis, NGS has helped healthcare workers diagnose and understand complex diseases [59, 60] at a much higher throughput than traditional methods and for many previously undiagnosed disease cases. NGS is now used in many thalassemia diagnostics [61–63]. In 2017, a targeted NGS technique was used to screen 951 minority Chinese Dai people in Yunnan for globin gene cluster carriers [64]. This method detected 49.5% of thalassemia carriers, compared to 22% using conventional methods. NGS is more accurate than conventional methods [65], especially for cases with normal red blood cell indices and hemoglobin fractions that conventional methods may miss [66]. NGS can simultaneously characterize known and unknown deletional and non-deletional mutations in all globin genes [58], reducing the likelihood of false-negative results, misdiagnoses, and the need for additional blood sampling and referral tests for thalassemia screening [67]. Furthermore, the current trend of combining NGS and PCR-based methods is gaining traction among researchers worldwide [63, 65]. Zhang and colleagues screened Chenzhou’s citizens for thalassemia variants using NGS and Gap-PCR in 2019. This combination found rare and novel thalassemia variants [63]. The combined method detects more variants, including rare, common, and novel ones. Thus, it improves at-risk couple detection at a low cost [68]. NGS has many benefits, but it requires expensive equipment and reagents, high sample throughput for pooled sequence analyses, and skilled bioinformaticians [62], whom LMICs lack. The proposed workflow for thalassemia genotyping is presented in Fig. 2.

## Global globin network

Under the umbrella of the international Human Variome Project [HVP; a UNESCO-partner organization, currently known as Global Variome and now a part of the Human Genome Organization (HUGO)], the Global Globin Network (GGN) is one of the priority initiatives, tackling hemoglobinopathies. The aim of GGN is to improve the quality and quantity of curated genomic data contributed by LMICs to internationally recognized genetic databases, in line with international best practices and in adherence to applicable ethical and regulatory frameworks and policies that assist and protect patients. Additionally, it aims to address the current “digital divide” that exists in the growing field of genomic medicine between HICs and LMICs. Hemoglobin disorders present a significant health problem in parts of the world where genomic technologies are particularly lacking. Thus, they represent an ideal entry point for these countries to develop the genetics and genomic medicine infrastructures and expertise necessary for the detection and reporting of globin variants as a basis for expansion into other areas of health-service delivery.

At the time of this writing, the GGN comprises 32 member countries, made up of 80 individual members including geneticists, hematologists, clinicians, bioinformaticians, and researchers working on hemoglobinopathies. The GGN has been expanded in order to include as many LMIC participants as possible. Of the 32 member countries, 21 are classified as LMICs and 11 as HICs. This composition supports the aim to bridge the marked gap between HICs and LMICs of genomic techniques employed for the diagnosis and management of hemoglobinopathies. The process envisaged to establish optimal disease management across the GGN is to pool knowledge globally, utilize resources synergistically, and integrate advanced diagnostic techniques in health systems to allow evidence-guided implementation of disease management and prevention programs. This is to ensure the achievement of a more uniform and effective approach and outcome, as these will be critically important for those GGN partners projected to experience a growing number of cases over the coming years, which might surpass the capacity of their health system. As a basis for all these efforts, from initial knowledge sharing to long-term training and collaboration, strengthening networking among the GGN partners is imperative. Importantly, since cutting-edge medical genetics and genomics methodologies already exist in most HICs, this can readily be employed to support development and growth in LMICs within the GGN and beyond.

To enlarge the network globally, GGN partnered with several international bodies, such as the Thalassaemia International Federation (TIF), the World Health Organization (WHO), the United Nations Educational, Scientific and Cultural Organization (UNESCO), the ClinGen Hemoglobinopathy Variant Curation Expert Panel [69], and the International Hemoglobinopathy Research Network (INHERENT). INHERENT is the most recent of GGN’s partnerships and sees the GGN participate as a large community within a network of overall over 200 experts from over 110 organizations, spanning 44 countries worldwide, to study genetic modifiers in hemoglobinopathies [70].

### Data collection and data sharing

To address the current digital divide amongst LMICs, and between LMICs and HICs, GGN recommended the Leiden Open Variation Database (LOVD) and the ITHANET Portal as platforms for data collection and data sharing. LOVD is designed to support the storage and sharing of genetic and phenotypic information for any kind of disease, so that based on the list of public LOVD installations (https://www.lovd.nl/3.0/public_list; accessed on 17 April 2024), there are currently 4 655 132 variants in 1 803 095 individuals held in 19 LOVD installations worldwide. By contrast, ITHANET (https://www.ithanet.eu/; accessed on 17 April 2024) is a web portal initially conceived specifically for hemoglobinopathies, which, as of this writing, holds in its databases sequence and functional annotation for 3462 variants in 538 genes. Details of 216 experts and 174 organizations working on hemoglobinopathies, epidemiological and healthcare information for 215 countries and regions, and HPLC information for 607 globin variants are also included. More recently, ITHANET launched new databases, namely IthaPhen [71] for genotype-phenotype correlation and IthaCNVs [72] for improved diagnosis of copy number variants in hemoglobinopathies.

Besides geographical epidemiological information, the establishment of databases holding ethnicity information is also important. Knowledge about prevalent variants in individual ethnic groups or communities can be used to streamline diagnostic procedures toward disease prevention and cost-effective health planning. In the area of genotype-phenotype correlation, interpretation of the clinical significance of variants novel to a clinical testing laboratory may be challenging; thus, sharing data among laboratories and standardizing representation are critically important [73]. Therefore, comprehensive coverage of all regions and ethnicities by genomic databases is instrumental for physicians and researchers around the world (i) to obtain information more speedily, (ii) to achieve cost-effective diagnosis and management of genetic disorders, and (iii) to provide improved insight into the causes, severity, and effect of common diseases. In addition, the establishment of, and conclusions derived from, genomic databases increase public awareness of the importance of genetics and genomics as key components of modern health care. This in turn will contribute to public health education as a critical pillar of any successful disease control program.

### The importance of establishing a genomic database for thalassemia

The distribution of thalassemia cases among ethnic groups varies according to the variant spectra and their frequencies across different geographical locations, ethnic groups, and population migrations. Thalassemia is an example of a typically recessive Mendelian disorder that is characterized by a distinct spectrum of globin mutations in different ethnic groups. Many of these may also carry characteristic polymorphisms in modifier genes, which are themselves not causative of disease but may at times have a profound impact on disease severity. The enormous diversity of causative β-globin mutations and modifier polymorphisms may be illustrated by using quick filters on ITHANET (www.ithanet.eu, accessed 17 April 2024). Accordingly, for the quick filter “Haemoglobinopathy/β-thalassaemia” on ITHANET, 491 variants were found that affect the *HBB* gene and cause β-thalassemia (https://www.ithanet.eu/db/ithagenes?action=list&hem=2), whereas a quick filter for “Functionality/Disease modifying mutations” revealed 886 entries, many of which are associated with changes in β-thalassemia disease severity (https://www.ithanet.eu/db/ithagenes?action=list&func=2).

The spectrum of causative and modifier variants may thus differ widely among ethnic groups due to different ancestral variants. Hence, it is necessary to have a database documenting at least all causative variants identified in a specific population, as a vital guide for designing assays and diagnostic tests for targeted detection of variants, and as a resource for the confirmation of diagnoses across diseases with similar symptoms [74].

There is a need for clinicians and scientists to explore variant information using multiple resources, such as publications and databases, to determine whether a suspected “newly discovered variant” has been previously characterized [75]. Ideally, instant access to any variant in a particular gene or locus of interest is required to conduct genomic research efficiently and to deliver “genetic healthcare” to the highest standards [76]. Obtaining up-to-date and accurate information on disease-causing variants is important in the diagnosis of conditions affecting human health. Therefore, a systematic collection of human variants, including genotype–phenotype correlation, is required to assist clinicians and genetic researchers in managing thalassemia globally.

The variants found during clinical testing could form an important regional resource for patient care, especially in LMICs, where information is sometimes not submitted in established databases. This may be due to some clinicians in LMICs being unaware of the existence of corresponding databases. Other reasons are lack of facilities, where laboratories may not be fully equipped to detect variants or may not have the required skills or IT facilities for submission of variants. Beyond technical insufficiency, further impediments to the submission of new variants are a lack of international communications, the absence of DNA bio-banking, restrictions by national authorities, and an inability to translate findings from original languages to English [77].

## Existing International Locus-specific Databases on thalassemia affiliated to Global Globin Network

### International locus-specific database

Based on various online resources and direct submissions of locus-specific databases (LSDBs), the Locus Specific Database list (https://grenada.lumc.nl/LSDB_list/lsdbs; accessed on 17 April 2024) reports 152 458 public LSDBs. Searching the genes that harbor the clinically most relevant thalassemia mutations, which are *HBB, HBA1*, and *HBA2*, several international LSDBs are included in the list. These databases are ClinVar, Global Variome shared LOVD, the Globin Gene Server/HbVar, the Malaysian Node of the Human Variome Project Database (MyHVPDb), and ITHANET. The establishment of international LSDBs is a mark of the success of LSDBs in harmonizing data and training database curators as one of the efforts to increase the quality and usefulness of LSDBs.

#### ITHANET

The IthaGenes database (https://www.ithanet.eu/db/ithagenes) [78] provides a graphical user interface with live and advanced search functionality and integrates and links gene and variant entries with other data on the ITHANET Portal and elsewhere. IthaGenes holds the most comprehensive dataset of mutations, deletions and copy number variations affecting the globin loci, with 2490 globin mutations (17 April 2024). However, the database also stores 886 disease-modifying mutations in 477 non-globin loci and 90 diagnostically relevant neutral polymorphisms, in acknowledgment of their diagnostic, prognostic, and therapeutic importance for hemoglobinopathies. Where appropriate, such as for epidemiological or chromatography data, data are also rendered graphically to aid comprehension and interpretation [78].

##### HbVar: a database of human hemoglobin variants and thalassemias

The HbVar database (https://globin.bx.psu.edu/hbvar/) was developed in 2001 by a multi-center academic effort and provides information on sequence variants for the globin genes and related thalassemias and hemoglobinopathies [79–81]. HbVar is a text-based locus-specific database with a total of 1873 database entries for globin mutations (17 April 2024), and with reference to an additional 37 modifier loci through a companion LOVD installation.

##### ClinGen & ClinVar

In 2013, a Clinical Genomic Resource (ClinGen) (www.clinicalgenome.org), a program under the auspices of the National Institutes of Health (NIH, USA), was launched. ClinGen was established as an authoritative central resource that defines the clinical relevance of genomic variants for use in precision medicine and research. The ClinVar database (www.ncbi.nlm.nih.gov/clinvar) is one of the first resources that was developed for ClinGen and as the primary site for deposition and retrieval of variant data and annotations provides freely accessible reports and supporting evidence linking somatic variants with clinical phenotypes [73, 82, 83]. It is maintained by the National Centre for Biotechnology Information (NCBI) and is supported by intramural National Institutes of Health (NIH) funding [73]. ClinVar is the present gold standard for variant databases, with a steadily growing coverage of variants and with emphasis on the reliability of reported disease association. The proposed thalassemia database will link to corresponding ClinVar entries, where these are available. Variants that are reported in ClinVar are determined by clinical testing laboratories, in contrast to the Human Gene Mutation Database (HGMD^®^) [84] and OMIM (www.omics.org/), which focus on variants documented in the literature [85]. Also, the variants reported in ClinVar include both germline and somatic variants, while COSMIC includes only somatic variants [86].

##### The Malaysian node of the human variome project database (MyHVPDb)

The Malaysian Node of the Human Variome Project Database (MyHVPDb, https://myhvpdb.kk.usm.my/genes; accessed on 17 April 2024) is an LSDB that stores human variants and phenotypes specific to Malaysia [87]. MyHVPDb was established in 2011 and collects, displays, and curates DNA variants in specific genes and phenotypes found in the Malaysian population, with emphasis on the genetic heterogeneity of the Malaysian ethnic groups. Based on LOVD 3.0 [88], MyHVPDb now integrates 3734 variants from 311 genes and 165 diseases, as collected from various databases, including PubMed, Scopus, and Google Scholar. MyHVPDb stores information on summary variant data, as well as full case-level information on individuals, phenotypes, screenings, and variants, with current limitation of clinical parameters to thalassemia disease. MyHVPDb has been configured to appear in the global listing of the LOVD central server in order to allow global data sharing and as a critical tool for data-guided disease management in Malaysia and in Malaysian or ethnically similar communities elsewhere.

### The case for multiple hemoglobinopathy data resources

ClinGen/ClinVar are the most reliable, genome-wide annotated resource for knowledge about disease causation by genetic variants. For a quickly expanding diagnostic landscape based on NGS, reliable annotation for the evaluation of variants detected across the genome is essential. However, this resource does not consider differences in disease severity or different phenotypic features for syndromic disorders, which is essential for specialists working on specific diseases, and it does not (currently) consider the influence of modifiers.

ITHANET with IthaGenes is the presently most comprehensive resource for hemoglobinopathies, which, beyond variant information for causative genes, also includes modifiers, detailed information on phenotypic features, detailed epidemiological information, national health policy and management information, and advanced tools for genotype-phenotype prediction for genetic counseling. For specialists working on hemoglobinopathies, this is an unrivalled and openly accessible resource. On the downside, it is not suitable for uncurated submissions and local installation for offline access.

LOVD is open-source, can be freely and easily installed locally in any environment (including environments off the grid or behind stringent firewalls), is widely used for many individual, LSDBs, is supported by frequent workshops, e.g. at the European Society of Human Genetics (ESHG) and HUGO meetings, and even allows installation of individual databases on a server in Leiden, the Netherlands, for those who cannot operate their own database server. It allows the storage of information for individual patients as well as automatic import of NGS data and synchronization with other LOVD databases. While the platform has limited support for same-disease information across loci, epidemiological information or integration of graphical information, such as HPLC chromatograms or maps, which are of great utility for hemoglobinopathies, it is an excellent choice for local database installations in low-resource environments, which nevertheless allows harmonization and synchronization with centralized databases. For this reason, it has been the choice for the Malaysian Node for the Human Variome Project Database and is the suggested platform for a central thalassemia database.

### Curation of the database using the LOVD platform

We recommend using LOVD 3 as it has offered many advantages for curators to manage LSDBs. This includes its flexibility for customization and extension, thus enabling the identification and addition of data items required for the extraction of a wide range of information at the user end.

LOVD 3 also offers five different access levels, i.e. administrator, manager, curator, submitter, and general user. Based on multiple access levels, it is easier to draw on a team of curators or expert advisors, which in turn facilitates data collection, verification, and submission from various centers and for dissemination of information to the general public. Furthermore, if necessary, the LOVD 3 system allows retrieval and transfer of the entire dataset to another suitable web-based application [89].

LOVD 3 was developed to connect with various resources, such as HGNC, NCBI, EBI, and Mutalyzer, as a basis for ongoing high-quality data provision. For instance, LOVD may draw on the NCBI sequence viewer to provide a visual representation of each database entry at its corresponding location and its interconnection with other NCBI resources [90]. The NCBI sequence viewer was selected over other genome browsers because of its simplicity and the ability to embed it in any page without the need for local installation and administration [91]. Moreover, LOVD allows links to original publications using the PubMed ID and/or DOI.

LOVD 3 provides different tables and linked tables, including individual, phenotype, screening, and variant(s). The “individual” table contains details on the family/patient studied, including gender, geographic origin, and patient identification as listed in the original publication. For thalassemia as a disease, the “phenotype” table includes the disease phenotypes as originally reported, which, in the case of the MyHVPDb, consists of clinical phenotype, clinical severity, age at thalassemia presentation, age at receiving the first blood transfusion, growth and development, hemoglobin level (g/l), the requirement for blood transfusion and spleen size (cm), all of which may be added to the database according to the clinical parameters of β-thalassemia patients. The “screening” table contains the detection template (DNA/RNA/Protein), techniques used, and genes screened. Meanwhile, the “variants” table includes information on allele, chromosome, genomic DNA change, published as, reference, dbSNP ID, frequency, and affects function.

It is essential to evaluate data quality during data collection, because entries may affect health decision-making, research, and clinical practice [92], and because the quality of content may differ widely depending on the responsible curator or contributor. Therefore, LOVD 3 was designed to fulfill this requirement, which may be one of the reasons why the customizable platform is a frequent point of reference for the Working Group for Variant Database Quality Assessment Criteria.

## Guidelines for genomic databases for thalassemia

**Figure 3.**
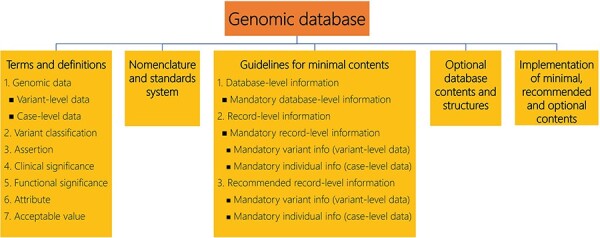
An overview of five key elements as the main core needed for the establishment of the thalassemia database. Adapted from [93].

This paper aims to provide the minimum requirements to enhance the contents of existing genomic databases in thalassemia that are based on the LOVD 3 platform (Supplementary Table S2). Therefore, it is well recognized that genomic databases for the annotation of genetic variants and phenotypes for thalassemia have great utilitarian value [92]; however, their inconsistencies across institutions (and more so countries) are of concern. Although not all the necessary information may be available for variants previously published in the literature, databases should at least be designed to include minimally required content. Figure 3 depicts an example of a genomic database with suggested minimal reporting of information for each case and variant observed, placing focus on material that can be accessed by the general public, while hiding more specific information that is restricted from access [93]. Around minimal content and structures for genomic databases, additional points need to be laid out in order to harmonize and facilitate ongoing contribution by multiple users, including terms and definitions, nomenclature and standards system, optional content and structures, and guidance on implementation with the database management software in question (Fig. 3). Adherence to these guidelines allows for usability but also great flexibility in the implementation of different databases of relevance for hemoglobinopathies, as listed in Table 1.

**Table 1.** Databases of thalassemia around the world

Databases	Diseases/Description	Link	References
ClinVar	Genetic diseases including thalassemia	https://www.ncbi.nlm.nih.gov/clinvar/	[73]
HbVar	Information about hemoglobin variants and mutations that cause thalassemia	https://globin.bx.psu.edu/hbvar/	[81]
Malaysian Node of the Human Variome Project Database (MyHVPDb)	Genetic diseases including thalassemia	https://myhvpdb.kk.usm.my	[87]
ITHANET	Thalassemia Sickle cell disease	https://www.ithanet.eu/	[106]

### Terms and definitions

The description of genomic data (variant- and case-level data), variant classification, assertion (of pathogenicity or otherwise), clinical significance, functional significance, attributes, and acceptance values are listed in the section of terms and definitions in the Supplementary Materials.

### Nomenclature and standards systems classification

As the required nomenclature system, gene names and variant descriptions ought to follow guidelines of the HUGO Gene Nomenclature Committee (HGNC) (https://www.genenames.org/) and HGVS Sequence Variant Nomenclature [94], respectively. Likewise, phenotype descriptions should consistently follow terms specified by Human Phenotype Ontology (HPO) (http://human-phenotype-ontology.github.io/), International Classification of Diseases (ICD, http://www.who.int/classifications/icd/en/), or International Classification of Diseases for Oncology (ICD-O, http://codes.iarc.fr/), depending on the focus of the database. However, the Online Mendelian Inheritance in Man (OMIM) (https://www.omim.org/) is a database used to define diseases. OMIM should be included with HPO in a genomic database to complement the information about the disease and phenotype [95]. For disease ontologies, guidelines from Orphanet Rare Disease Ontology (ORDO) [96], Mondo Disease Ontology (MONDO) [97], and Dictionary of Disease Ontologies (DODO) [98] are recommended. Besides, for recommended standards, a number of variant classification standards have been created, as detailed under Terms and Definitions.

### Guidelines for minimal contents

Depending on the deployed portal or database system, the minimally required contents can be separated into two distinct categories: database-level information and record-level information (Fig. 3).

#### Mandatory database-level information

The following details must be presented on the website for the database, as detailed elsewhere [99, 100]. For thalassemia genomic variant databases per gene, there are a few vital elements that should be focused on, including the use of HGNC Gene symbols, HGNC IDs, OMIM, and HGNC-approved names. Reference sequences of the genome must be based on a build of the genome that is up-to-date and well-defined (please refer to http://varnomen.hgvs.org for details). In other circumstances, suitable RefSeq, chromosomal/genomic, transcript, and protein sequences should be referenced [101]. Any deviation from reference should be described in detail, and the reference sequence used for variant descriptions in the database should be specified. This information is generally available in LOVD via the RefSeq URL link on the gene homepage. Lastly, a specification for multivariant allele and/or aberrant sequence variant nomenclature may be added in addition to the obligatory HGVS sequence variant nomenclature. Any relevant information on imprinting, which influences gene expression and thus illness or trait inheritance, should be presented. Such information helps prevent problems in the interpretation of RNA-based studies, such as mistaken assumptions of homozygosity or difficulties explaining the absence of DNA-level variations at the RNA level. As additional details, information and/or stable connections to other online resources (such as GeneReviews; https://www.ncbi.nlm.nih.gov/books/NBK1116/ and Genetics Home Reference; https://ghr.nlm.nih.gov/) provide access to further biological and medical information that may be valuable to the user community.

#### Mandatory record-level information

Mandatory variant information (variant-level data) and mandatory individual information (case-level data) are the minimally required data for genomic variant databases and both are vital to reporting variations and phenotypes.

##### Mandatory variant information (variant-level data)

For the mandatory variant information (variant-level data), there are a few elements of key concern. First, high-throughput sequencing (next-generation sequencing, NGS) is becoming increasingly important in the field of DNA diagnostics and extends beyond individual loci, so that variants need to be described at the chromosomal level, for example, NC_000023.11:g.115641445_115653674del. Researchers from LMICs can benefit from NGS and gain experience with the platform through collaboration with their counterparts in HICs, which might take the form of academic collaboration or, more controversially, of business investment in future markets for diagnostic and medical products. The increased disease burden in HICs by migration from LMICs may also create incentives long-term for HICs to invest in training, funding, and logistics support for NGS in LMICs, similar to what is being proposed for the control of other global health challenges, such as antibiotic resistance [102]. Because of this, bioinformatics pipelines can leverage HGVS genomic DNA variant descriptions, which are very straightforward, in database searches and (automated) submissions. Using chromosomal-level descriptions eliminates errors caused by improper HGVS coding DNA sequence variant nomenclature in NGS pipeline variant callers. The description of every coding DNA sequence on a chromosome is redundant from the genomics perspective, which concentrates on chromosomal-level descriptions. In addition, at the level of the RNA transcript, a variant is described as an example, e.g. NM_003001.3:r. = (no effect), NM_003001.3:r.(23dup)(predicted), NM_003001.3:r.23dup (RNA analyzed). When describing a variant at the RNA and coding DNA levels, the same transcript reference sequence is used, but with a different prefix. The HGVS nomenclature demands predicted consequences have to be reported in parentheses “()” as an example; NM_003001.3:r.(23dup). The goal of describing a variant at the RNA level is to show how the variant located at the coding DNA level is projected or spotted to modify the resulting transcript. It is important to report all possible products resulting from the partial effects on splicing, whether they have been examined or prognosticated. Furthermore, the variant at the coding DNA level [e.g. NM_003001.3:c.(23dup)] is described using the transcript reference sequence in compliance with HGVS nomenclature criteria (see http://varnomen.hgvs.org/bg-material/refseq/). The MANE transcript for a gene should always be used for the reporting of variants on the transcript level. When variants are reported in relation to a transcript, the preferred reference sequence is the reference suggested by the Matched Annotation from the NCBI and EMBL-EBI (MANE) project. The main goal of this explanation is to raise awareness regarding the possibility of incorrectly interpreting variant effects at the transcript and protein levels due to aberrant splicing caused by the observed variant. At the level of the protein, a variant is described as follows: NP_002992.1, e.g. NP_002992.1:p.(His8Glnfs*12). The parental origin of the variant also needs to be described. This information is critical for both recessive and imprinted disorders, as well as for the evaluation of *de novo* variants in autosomal dominant disorders. Techniques such as SEQ (sequencing), MLPA (multiplex ligation-dependent probe amplification), SEQ-NG (NGS), PTT (protein truncation test), and array SNP are used to detect and validate the sequence variant. In addition, all molecule types (unknown, DNA, RNA, or protein) used to detect or confirm the reported variation instance should also be specified. This information and the methodologies employed are intended to facilitate data quality assessment, while also providing other institutions with guidance when implementing similar testing/investigations of their patients. Finally, each variant should be assigned a unique and permanent identifier, to allow unambiguous reference, and in turn a link to the original information source should be provided, such as a publication, submitter, or central database.

##### Mandatory individual information (case-level data)

Mandatory individual information (case-level data) includes a unique submitter ID (preferably ORCID) and a pseudonymized, potentially non-public lab-ID code for each analyzed individual, so that the individual cannot readily be identified from the data. Each case-level record in the database also needs to have a consistent, unique, and permanent identifier. Besides, for individuals with a disease phenotype, the phenotype is reported, whereas for pre-symptomatic or carrier individuals the phenotype of an affected family member is reported; additionally, the genotype of unaffected family members is given. In addition, the list of potential genes (for a certain disease or trait) that have been evaluated in a person, without detecting variations, should also be reported. Importantly, case-level data will be essential for the recording of phenotypic variability typical of hemoglobinopathies. It will also record any variants detected in known disease modifiers to allow an analysis of their influence on the phenotype across databases/across all case-level data in the centralized database.

#### Recommended record-level information

It is recommended that the contents of genetic variant and disease-specific databases be expanded as described below for a more comprehensive evaluation of the clinical effects of variants. Additionally, the specified fields and their contents for genetic variation databases can be classified into variant-level and case-level data.

##### Recommended variant information (variant-level data)

Several details should be highlighted for the recommended variant information (variant-level data). First, the variant at the level of gene- or region-specific DNA needs to be described, regarding which one can refer to the HGVS sequence variant nomenclature (http://varnomen.hgvs.org/) for more information, permitted values, and further examples. The functional consequence (functional significance) such as descriptions: not known to affect function (-), probably not affecting function (-?), unknown (?), probably affects function (+?), affects function (+), needs to be included as well. Ideally, these descriptions are accompanied by a reference to the source of the claim (submitter, curator, expert panel, functional database, PubMed ID). Besides, the number of times the variant allele was reported is also important to include, especially for variants that have functional effects [such as 1 = found in one patient only, 12 = found in 12 individuals (patient and other family members)]. It is recommended to include the number of probands separately from family members. It is also necessary to provide information about the genetic origin of the variant, such as germline, somatic, *de novo*, (unspecified, maternal, or paternal) uniparental disomy, and *in vitro* (cloned for functional testing). Additionally, it is critical to include information about the tissue sample(s) used to find the variant, comments on the described variant, links to population frequency databases, the number of exons/introns affected by the variation, and the frequency with which the variant allele is reported. Furthermore, the description of the variant as it might have appeared in the original publication, its common name/alias, and the phenotypic segregation of the variation are also important. Variant-phenotype segregations, such as descriptions: ? = unknown, yes = segregates with the disease, no = does not segregate with the disease, even more significant if providing the number of segregations or non-segregations, exon and intron numbers affected by the variation (as an example, 01 or 01e = exon 1, 02i = intron 2, 03_07 = exons 3 to 7) and links to databases of population frequency, are also crucial to introduce in the variant-level data section. Lastly, the assertion needs to be provided. For further information, please refer to the section on terms and definitions in the supplementary materials. If different variants cause different diseases, the clinical assertion should also include the disease against which the assertion is made. It would be preferable to include a description of the evidence used in the clinical assertion of the variant, as well as references.

##### Recommended individual information (case-level data)

There are a few important components that should be considered for the recommended individual information (case-level data). First, the disease’s name in other databases or elsewhere needs to be mentioned. A list of the genes tested in the individual also needs to be included. In the case of exome sequencing, for example, a link or reference to the list of genes included in a capture kit is sufficient. It is also worth mentioning whole-genome sequencing, if done. The details such as age at diagnosis, gender of the person, consanguinity of the parents’ marriages, the number of patients reported in a family or population, the individual’s geographical/ancestral origin, remarks about the person, extended phenotypic information about the person tested, and the inheritance pattern of the disease or trait in the individual’s family should be included. Finally, the name/alias of the disease in other databases should be included, and the individual population should preferably be based on self-assessed indigenous groups as well.

### Optional database contents and structure

Several recommendations could be explored to enhance the utility of databases:

1. Storing variant combinations (alleles and genotypes) observed in each gene (investigated) per individual. In theory, databases can employ the HGVS allele or genotype description format; however, this is rarely done to avoid variant search and sort issues. This includes linking all *cis* or *trans* variants found in the individual’s record—combining this information with each individual’s health or disease state will support key conclusions about the impact of variations, especially those responsible for recessive disorders or detected in critical genes or those that may modify the phenotype.
2. Storing all gene variant data from an individual, especially variants of unclear significance, allows for frequency data collection, which aids in classification judgments (or even future analysis pertinent to genetic modifiers of the disease phenotype).
3. Comments should be enabled, with ID tracking for submissions and edits by registered users, who after all are the experts in the topic and are frequently the database curators.
4. The addition of functional assays and detailed phenotypes to genetic variation databases may transform them into knowledge bases, given the availability of appropriate search and interpretation tools.
5. It is anticipated that links to precise phenotypes, pedigree information, time course changes, and other identifiable information in specialized patient and disease registries with minimally restricted access regulations will boost the clinical usefulness of these databases.

### Implementation of minimal, recommended, and optional content

In genomic variant databases that use the LOVD platform (https://www.lovd.nl/), additional information may be found about the contents of the various database fields and examples of how they may be implemented or expanded (Fig. 3). We also propose the list of informative phenotypic data for thalassemia patients, which can be managed in LOVD, by referring to Supplementary Table S1.

### Toward an instance of the thalassemia database

From concept to concrete database, the GGN and the Malaysian HVP node have been co-organizing workshops for the implementation of LOVD-based local databases for hemoglobinopathies and their connection to central database installations in Leiden. As GGN member countries are making efforts to achieve funding for such implementations, education continues to cover essential practical, safety, ethical, and quality aspects for the envisaged database. This includes considerations for the establishment of (local) database instances, such as that it be non-redundant with existing resources, that long-term funding is available for maintenance, that a common data model is applied, allowing exchange of data, and that its data can readily contribute by synchronization or other means to the centralized Thalassemia database [103]. Tied in with this are aspects of quality for each database, which goes from (i) data quality, e.g. by measures to reduce clerical errors and ensure accountability, over (ii) technical quality relating to the quality of the platform, control measures, and speed, over (iii) timeliness concerning frequent data updates, to (iv) accessibility, which includes adequate documentation, searchability, and usability of the platform interface [80]. Such training further covers the awareness and adherence to high curation standards to turn each local database and thus the central resource into a meaningful tool for the community [104], and likewise awareness and means of maintaining confidentiality and privacy of data through suitable security and pseudonymization procedures in local storage and in the national and international sharing of data [105].

## Conclusion and recommendations

In 2015, the Human Variome Project, a worldwide non-governmental organization, launched GGN with a focus on thalassemias and hemoglobinopathies. Since then, the GGN has grown rapidly and attracted many participants from LMICs endemic for these disorders. Its focus is on hemoglobinopathies as a gateway to genomic medicine in low-resource environments and as a proof of concept for the case of international sharing of hemoglobin variant information, enabling expertise and knowledge sharing among the involved countries and spurring a variety of research projects in the field. One of its major initiatives is spearheading the creation of global thalassemia and hemoglobinopathy databases and resolving the challenging technical aspects of its development, while maintaining close collaboration with existing regional or country-based databases. This database has an additional benefit over heterogeneous patient-centric registries, in that the data are anonymous, allowing for more extensive worldwide data exchange with fewer restrictions for various clinical and academic applications. The database is also aligned with the variant classification using the ACMG/AMP guidelines. Additionally, it has identified a collaborative network of experts who are eager to participate in the different facets of variant sharing between nations and who are sensitive to meeting the needs of diverse populations. The establishment of individual LOVD-based variant databases in GGN member countries and their integration with the ITHANET Portal as a central resource for hemoglobinopathies as part of this effort may be instrumental in initiating essential data-guided disease management in LMICs and in capturing the diversity of hemoglobinopathies as an international resource.

## Supplementary Material

baae080_Supp

## References

[1] Kleanthous M , Phylactides M. Thalassemia and its relevance to personalized medicine. *Pers Med* 2008;5:141–53. doi: 10.2217/17410541.5.2.141 29783353

[2] Kumar R , Sagar C, Sharma D et al. β-globin genes: mutation hot-spots in the global thalassemia belt. *Hemoglobin* 2015;39:1–8. doi: 10.3109/03630269.2014.985831 25523871

[3] Cao A , Kan YW. The prevention of thalassemia. *Cold Spring Harb Perspect Med* 2013;3:a011775. 10.1101/cshperspect.a011775 PMC3552345 23378598

[4] Shafie AA , Wong JH, Ibrahim HM et al. Economic burden in the management of transfusion-dependent thalassaemia patients in Malaysia from a societal perspective. *Orphanet J Rare Dis* 2021;16:157. doi: 10.1186/s13023-021-01791-8 PMC8028190 33827621

[5] Eleftheriou A , Angastiniotis M. Global thalassaemia review 2021. Thalassaemia International Federation. 2021.

[6] Weatherall DJ . The challenge of haemoglobinopathies in resource-poor countries. *Br J Haematol* 2011;154:736–44. doi: 10.1111/j.1365-2141.2011.08742.x 21726207

[7] Halim-Fikri BH , Lederer CW, Baig AA et al. Global globin network consensus paper: classification and stratified roadmaps for improved thalassaemia care and prevention in 32 countries. *J Pers Med* 2022;12:552. doi: 10.3390/jpm12040552 PMC9032232 35455667

[8] Kattamis A , Forni GL, Aydinok Y et al. Changing patterns in the epidemiology of β‐thalassemia. *Eur J Haematol* 2020;105:692–703. doi: 10.1111/ejh.13512 32886826 PMC7692954

[9] Ibrahim HM , Muda Z, Othman IS et al. Observational study on the current status of thalassaemia in Malaysia: a report from the Malaysian Thalassaemia Registry. *BMJ Open* 2020;10:e037974. doi: 10.1136/bmjopen-2020-037974 PMC7328811 32601117

[10] De Sanctis V , Kattamis C, Canatan D et al. β-thalassemia distribution in the old world: an ancient disease seen from a historical standpoint. *Mediterr J Hematol Infect Dis* 2017;9:e2017018. doi: 10.4084/MJHID.2017.018 PMC5333734 28293406

[11] Dhamcharee V , Romyanan O, Ninlagarn T. Genetic counseling for thalassemia in Thailand: problems and solutions. *Southeast Asian J Trop Med Public Health* 2001;32:413–18. 11556598

[12] Fleming KA Naidoo M Wilson M et al. High-quality diagnosis: an essential pathology package. In: Jamison DT, Gelband H and Horton S*et al*. (eds), *Disease Control Priorities: Improving Health and Reducing Poverty*, 3rd edn. Washington DC: The International Bank for Reconstruction and Development/The World Bank, 2017. 30212155

[13] Tritipsombut J , Sanchaisuriya K, Phollarp P et al. Micromapping of thalassemia and hemoglobinopathies in different regions of northeast Thailand and Vientaine, Laos People’s Democratic Republic. *Hemoglobin* 2012;36:47–56. doi: 10.3109/03630269.2011.637149 22122810

[14] Premawardhana AP , Mudiyanse R, De Silva ST et al. A nationwide survey of hospital-based thalassemia patients and standards of care and a preliminary assessment of the national prevention program in Sri Lanka. *PloS One* 2019;14:e0220852. doi: 10.1371/journal.pone.0220852 PMC6697367 31419232

[15] Hossain MS , Raheem E, Sultana TA et al. Thalassemias in South Asia: clinical lessons learnt from Bangladesh. *Orphanet J Rare Dis* 2017;12:93. doi: 10.1186/s13023-017-0643-z PMC5437604 28521805

[16] Jo C . Cost-of-illness studies: concepts, scopes, and methods. *Clin Mol Hepatol* 2014;20:327–37. doi: 10.3350/cmh.2014.20.4.327 25548737 PMC4278062

[17] Jefferson T , Demichelli V, and Mugford M. *Elementary Economic Evaluation in Health Care*. London: BMJ Publications, 2000.

[18] Reed-Embleton H , Arambepola S, Dixon S et al. A cost-of-illness analysis of β-Thalassaemia major in children in Sri Lanka–experience from a tertiary level teaching hospital. *BMC Pediatric* 2020;20:257. doi: 10.1186/s12887-020-02160-3 PMC7251920 32460774

[19] Kantharaj A , Chandrashekar S. Coping with the burden of thalassemia: aiming for a thalassemia free world. *Global J Transf Med* 2018;3:1–5. doi: 10.4103/GJTM.GJTM_19_18

[20] Esmaeilzadeh F , Azarkeivan A, Emamgholipour S et al. Economic burden of thalassemia major in Iran, 2015. *J Health Sci Res* 2016;16:111–15. PMC7191027 27840337

[21] Riewpaiboon A , Nuchprayoon I, Torcharus K et al. Economic burden of beta-thalassemia/Hb E and beta-thalassemia major in Thai children. *BMC Res Notes* 2010;3:29. doi: 10.1186/1756-0500-3-29 PMC2835719 20181056

[22] Azman NF , Abdullah WZ, Mohamad N et al. Practice of iron chelation therapy for transfusion-dependent thalassemia in Southeast Asia. *Asian Biomed* 2016;10:537–47. doi: 10.5372/1905-7415.1006.524

[23] Safdar S , Mirbahar A, Sheikh MA et al. Economic burden of thalassemia on parents of thalassemic children: a multi-centre study. *Pak J Med Res* 2017;56:68–72.

[24] Koren A , Profeta L, Zalman L et al. Prevention of β thalassemia in Northern Israel-A cost-benefit analysis. *Mediterr J Hematol Infect Dis* 2014;6:e2014012. doi: 10.4084/MJHID.2014.012 PMC3965716 24678389

[25] Colah R , Italia K, Gorakshakar A. Burden of thalassemia in India: the road map for control. *Pediatr Hematol Oncol J* 2017;2:79–84. doi: 10.1016/j.phoj.2017.10.002

[26] Yadav SS , Panchal P, Menon KC. Prevalence and management of beta-thalassemia in India. *Hemoglobin* 2022;46:27–32. doi: 10.1080/03630269.2021.2001346 35129043

[27] Moirangthem A , Phadke SR. Socio-demographic profile and economic burden of treatment of transfusion dependent thalassemia. *Indian J Pediatr* 2018;85:102–07. doi: 10.1007/s12098-017-2478-y 29119463

[28] Chandy M . Developing a national programme for India. In: Ghosh K and Colah R (eds), *Control and Management of Thalassemia and Other Hemoglobinopathies in the Indian Subcontinent_ Synoptic Views*. Mumbai: National Institute of Immunohaematology, 2008, 46e9.

[29] Sinha S , Seth T, Colah RB et al. Haemoglobinopathies in India: estimates of blood requirements and treatment costs for the decade 2017–2026. *J Community Genet* 2020;11:39–45. doi: 10.1007/s12687-019-00410-1 30756298 PMC6962406

[30] Rao TS , Prabhakar AK, Jagannatha Rao KS et al. Relationship between consanguinity and depression in a south Indian population. *Indian J Psychiatry* 2009;51:50–52. doi: 10.4103/0019-5545.44906 19742204 PMC2738415

[31] Singh P , Shaikh S, Parmar S et al. Current status of β-thalassemic burden in India. *Hemoglobin* 2023;47:181–90. doi: 10.1080/03630269.2023.2269837 37947120

[32] Saxena A , Phadke SR. Feasibility of thalassaemia control by extended family screening in Indian context. *J Health Popul Nutr* 2002;20:31–35. 12022156

[33] Garg R , Agrawal P, Malhotra N et al. Thalassemia: an Indian perspective. *World J Anemia* 2018;2:11–15.

[34] Rees DC , Brouse VAM. Sickle cell disease: status with particular reference to India (Editorial). *Ind J Med Res* 2016;143:675–77. doi: 10.4103/0971-5916.191916 PMC5094104 27748289

[35] Nigam N Singh PK Bhatnagar S et al. An early diagnosis of thalassemia: a boon to a healthy society. In: Aise Seda A (ed.), *Blood - Updates on Hemodynamics and on Thalassemia*. Internet: IntechOpen, 2022.

[36] Kountouris P , Michailidou K, Christou S et al. Effect of HBB genotype on survival in a cohort of transfusion-dependent thalassemia patients in Cyprus. *Haematologica* 2021;106:2458–68. doi: 10.3324/haematol.2020.260224 32732363 PMC8409026

[37] Huang TL , Zhang TY, Song CY et al. Gene mutation spectrum of thalassemia among children in Yunnan province. *Front. Pediatr* 2020;8:159. doi: 10.3389/fped.2020.00159 PMC7174584 32351918

[38] Lin M , Zhu JJ, Wang Q et al. Development and evaluation of a reverse dot blot assay for the simultaneous detection of common alpha and beta thalassemia in Chinese. *Blood Cells Mol Dis* 2012;48:86–90. doi: 10.1016/j.bcmd.2011.12.001 22197394

[39] Bilgen T , Clark ÖA, Öztürk Z et al. Gap-PCR screening for common large deletional mutations of β-globin gene cluster revealed a higher prevalence of the Turkish inversion/deletion (δβ) 0 mutation in antalya. *Turkish J Hematol* 2016;33:107. doi: 10.4274/tjh.2014.0242 PMC5100720 26377447

[40] Hu S , Zhan W, Wang J et al. Establishment and application of a novel method based on single nucleotide polymorphism analysis for detecting β-globin gene cluster deletions. *Sci Rep* 2020;10:1–9. doi: 10.1038/s41598-020-75507-6 33106596 PMC7588424

[41] El-Gawhary S , El-Shafie S, Niazi M et al. Study of β-thalassemia mutations using the polymerase chain reaction-amplification refractory mutation system and direct DNA sequencing techniques in a group of Egyptian thalassemia patients. *Hemoglobin* 2007;31:63–69. doi: 10.1080/03630260601057104 17365006

[42] Zhuang J , Tian J, Wei J et al. Molecular analysis of a large novel deletion causing α+-thalassemia. *BMC Med Genet* 2019;20:74. doi: 10.1186/s12881-019-0797-8 PMC6501318 31060505

[43] Yuregir OO , Ayaz A, Yalcintepe S et al. Detection of α-Thalassemia by using multiplex ligation-dependent probe amplification as an additional method for rare mutations in southern Turkey. *Indian J Hematol Blood Transfus* 2016;32:454–59. doi: 10.1007/s12288-015-0617-z 27812256 PMC5074955

[44] Liu JZ , Han H, Schouten JP et al. Detection of α-thalassemia in China by using multiplex ligation-dependent probe amplification. *Hemoglobin* 2008;32:561–71. doi: 10.1080/03630260802508111 19065334

[45] Jomoui W , Srivorakun H, Chansai S et al. Loop-mediated isothermal amplification (LAMP) colorimetric phenol red assay for rapid identification of α0-thalassemia: application to population screening and prenatal diagnosis. *PloS One* 2022;17:e0267832. doi: 10.1371/journal.pone.0267832 PMC9049341 35482800

[46] Tepakhan W , Jomoui W. Rapid molecular diagnostics of large deletional β0-thalassemia (3.5 kb and 45 kb) using colorimetric LAMP in various thalassemia genotypes. *Heliyon* 2021;7:e08372. doi: 10.1016/j.heliyon.2021.e08372 PMC8591500 34816050

[47] Peng C , Zhang H, Chen H et al. Analysis of rare thalassemia genetic variants based on third generation sequencing. *Res Square* 2022;12:9907. doi: 10.1038/s41598-022-14038-8 PMC9197973 35701592

[48] Wong C , Dowling CE, Saiki RK et al. Characterization of β-thalassaemia mutations using direct genomic sequencing of amplified single copy DNA. *Nature* 1987;330:384–86. doi: 10.1038/330384a0 3683554

[49] Hussein G , Fawzy M, El Serafi T et al. Rapid detection of β-thalassemia alleles in Egypt using naturally or amplified created restriction sites and direct sequencing: a step in disease control. *Hemoglobin* 2007;31:49–62. doi: 10.1080/03630260601057088 17365005

[50] Losekoot M , Fodde R, Harteveld CL et al. Homozygous beta+ thalassaemia owing to a mutation in the cleavage-polyadenylation sequence of the human beta globin gene. *J Med Genet* 1991;28:252–55. doi: 10.1136/jmg.28.4.252 1856830 PMC1016827

[51] Losekoot M , Fodde R, Harteveld CL et al. Denaturing gradient gel electrophoresis and direct sequencing of PCR amplified genomic DNA: a rapid and reliable diagnostic approach to beta thalassaemia. *Br J Haematol* 1990;76:269–74. doi: 10.1111/j.1365-2141.1990.tb07883.x 2094329

[52] Pooladi N , Hosseinpour Feizi MA, Haghi M et al. Analysis of beta thalassemia mutations using the single strand conformation polymorphism (SSCP) technique. *Sci J Kurdistan Univ Med Sci* 2010;15:13–19.

[53] Li W , Gao F, Tang W et al. Detection of known thalassemia point mutations by snapback single-strand conformation polymorphism: the feasibility analysis. *Clin Biochem* 2006;39:833–42. doi: 10.1016/j.clinbiochem.2006.05.004 16820146

[54] Quek DL , Ng YY, Wang W et al. Rapid carrier screening for β-thalassemia by single-step allele-specific PCR and detection. *Clin Biochem* 2007;40:427–30. doi: 10.1016/j.clinbiochem.2007.01.003 17296174

[55] Fanis P , Kousiappa I, Phylactides M et al. Genotyping of BCL11A and HBS1L-MYB SNPs associated with fetal haemoglobin levels: a SNaPshot minisequencing approach. *BMC Genomics* 2014;15:1–2. doi: 10.1186/1471-2164-15-108 24502199 PMC3922441

[56] Sajadpour Z , Amini-Farsani Z, Motovali-Bashi M et al. Investigation of RFLP haplotypes β-globin gene cluster in beta-thalassemia patients in central Iran. *Int J Hematol Oncol Stem Cell Res* 2019;13:61–67. 31372199 PMC6660478

[57] Achour A , Koopmann TT, Baas F et al. The evolving role of next-generation sequencing in screening and diagnosis of hemoglobinopathies. *Front Physiol* 2021;12:686689. doi: 10.3389/fphys.2021.686689 PMC8353275 34385932

[58] Sabath DE . Molecular diagnosis of thalassemias and hemoglobinopathies: an ACLPS critical review. *Am J Clin Pathol* 2017;148:6–15. doi: 10.1093/ajcp/aqx047 28605432

[59] Yang Y , Muzny DM, Reid JG et al. Clinical whole-exome sequencing for the diagnosis of mendelian disorders. *N Engl J Med* 2013;369:1502–11. doi: 10.1056/NEJMoa1306555 24088041 PMC4211433

[60] Stark Z , Tan TY, Chong B et al. A prospective evaluation of whole-exome sequencing as a first-tier molecular test in infants with suspected monogenic disorders. *Genet Med* 2016;18:1090–96. doi: 10.1038/gim.2016.1 26938784

[61] He S , Qin Q, Yi S et al. Prevalence and genetic analysis of α-and β-thalassemia in Baise region, a multi-ethnic region in southern China. *Gene* 2017;619:71–75. doi: 10.1016/j.gene.2016.02.014 26877226

[62] Shang X , Peng Z, Ye Y et al. Rapid targeted next-generation sequencing platform for molecular screening and clinical genotyping in subjects with hemoglobinopathies. *EBioMed* 2017;23:150–59. doi: 10.1016/j.ebiom.2017.08.015 PMC5605365 28865746

[63] Zhang H , Li C, Li J et al. Next‐generation sequencing improves molecular epidemiological characterization of thalassemia in Chenzhou region, PR China. *J Clin Lab Analysis* 2019;33:e22845. doi: 10.1002/jcla.22845 PMC6528559 30809867

[64] He J , Song W, Yang J et al. Next-generation sequencing improves thalassemia carrier screening among premarital adults in a high prevalence population: the Dai nationality, China. *Genet Med* 2017;19:1022–31. doi: 10.1038/gim.2016.218 28125089

[65] Munkongdee T , Chen P, Winichagoon P et al. Update in laboratory diagnosis of thalassemia. *Front Mol Biosci* 2020;7:74. doi: 10.3389/fmolb.2020.00074 PMC7326097 32671092

[66] Alauddin H , Jaapar NA, Azma RZ et al. A case series of α-thalassemia intermedia due to compound heterozygosity for Hb Adana [HBA2: c179G> A (or HBA1); p. Gly60Asp] with other α-thalassemias in Malay families. *Hemoglobin* 2014;38:277–81. doi: 10.3109/03630269.2014.916720 24829075

[67] Vijian D , Ab Rahman WS, Ponnuraj KT et al. Molecular detection of alpha thalassemia: a review of prevalent techniques. *Medeniyet Med J* 2021;36:257–69. doi: 10.5222/MMJ.2021.14603 PMC8565582 34915685

[68] Zhao J , Li J, Lai Q et al. Combined use of gap-PCR and next-generation sequencing improves thalassaemia carrier screening among premarital adults in China. *J Clin Pathol* 2020;73:488–92. doi: 10.1136/jclinpath-2019-206339 31980563 PMC7398480

[69] Kountouris P , Stephanou C, Lederer CW et al. Adapting the ACMG/AMP variant classification framework: a perspective from the ClinGen hemoglobinopathy variant curation expert panel. *Human Mutation* 2022;43:1089–96. doi: 10.1002/humu.24280 34510646 PMC9545675

[70] Kountouris P , Stephanou C, Archer NM et al. The International Hemoglobinopathy Research Network (INHERENT): an international initiative to study the role of genetic modifiers in hemoglobinopathies. *Blood* 2021;138:948. doi: 10.1002/ajh.26323 PMC10390849 34406671

[71] Xenophontos M , Minaidou A, Stephanou C et al. IthaPhen: an interactive database of genotype-phenotype data for hemoglobinopathies. *Hemasphere* 2023;7:e922. doi: 10.1097/HS9.0000000000000922 PMC10289560 37359188

[72] Minaidou A , Tamana S, Stephanou C et al. A novel tool for the analysis and detection of copy number variants associated with haemoglobinopathies. *Int J Mol Sci* 2022;23:15920. doi: 10.3390/ijms232415920 PMC9782104 36555557

[73] Landrum MJ , Lee JM, Benson M et al. ClinVar: public archive of interpretations of clinically relevant variants. *Nucleic Acids Res* 2016;44:D862–8. doi: 10.1093/nar/gkv1222 26582918 PMC4702865

[74] Tan EC , Loh M, Chuon D et al. Singapore human mutation/polymorphism database: a country‐specific database for mutations and polymorphisms in inherited disorders and candidate gene association studies. *Human Mutation* 2006;27:232–35. doi: 10.1002/humu.20291 16429432

[75] Park MH , Koo SK, Lee JS et al. KMD: Korean mutation database for genes related to diseases. *Human Mutation* 2012;33:E2332–40. doi: 10.1002/humu.22039 22323337

[76] Cotton RG , Auerbach AD, Brown AF et al. A structured simple form for ordering genetic tests is needed to ensure coupling of clinical detail (phenotype) with DNA variants (genotype) to ensure utility in publication and databases. *Human Mutation* 2007;28:931–32. doi: 10.1002/humu.20631 17726697

[77] Auerbach AD , Burn J, Cassiman JJ et al. Mutation (variation) databases and registries: a rationale for coordination of efforts. *Nat Rev Genet* 2011;12:881. doi: 10.1038/nrg3011-c1 22025002

[78] Kountouris P , Lederer CW, Fanis P et al. IthaGenes: an interactive database for haemoglobin variations and epidemiology. *PloS One* 2014;9:e103020. doi: 10.1371/journal.pone.0103020 PMC4109966 25058394

[79] Giardine B , Borg J, Viennas E et al. Updates of the HbVar database of human hemoglobin variants and thalassemia mutations. *Nucleic Acids Res* 2014;42:D1063–9. doi: 10.1093/nar/gkt911 24137000 PMC3964999

[80] Giardine B , van Baal S, Kaimakis P et al. HbVar database of human hemoglobin variants and thalassemia mutations: 2007 update. *Human Mutation* 2007;28:206. doi: 10.1002/humu.9479 17221864

[81] Hardison RC , Chui DH, Giardine B et al. HbVar: a relational database of human hemoglobin variants and thalassemia mutations at the globin gene server. *Human Mutation* 2002;19:225–33. doi: 10.1002/humu.10044 11857738

[82] Landrum MJ , Lee JM, Riley GR et al. ClinVar: public archive of relationships among sequence variation and human phenotype. *Nucleic Acids Res* 2014;42:D980–5. doi: 10.1093/nar/gkt1113 24234437 PMC3965032

[83] Rehm HL , Berg JS, Brooks LD et al. ClinGen. ClinGen—the clinical genome resource. *New Engl J Med* 2015;372:2235–42. doi: 10.1056/NEJMsr1406261 26014595 PMC4474187

[84] Stenson PD , Mort M, Ball EV et al. The human gene mutation database: towards a comprehensive repository of inherited mutation data for medical research, genetic diagnosis and next-generation sequencing studies. *Hum Genet* 2017;136:665–77. doi: 10.1007/s00439-017-1779-6 28349240 PMC5429360

[85] Landrum MJ , Kattman BL. ClinVar at five years: delivering on the promise. *Human Mutation* 2018;39:1623–30. doi: 10.1002/humu.23641 30311387 PMC11567647

[86] Forbes SA , Beare D, Boutselakis H et al. COSMIC: somatic cancer genetics at high-resolution. *Nucleic Acids Res* 2017;45:D777–83. doi: 10.1093/nar/gkw1121 27899578 PMC5210583

[87] Hashim AH , Etemad A, Latif AZ et al. The first Malay database toward the ethnic-specific target molecular variation. *BMC Res Notes* 2015;8:176. doi: 10.1186/s13104-015-1123-y PMC4440489 25925844

[88] Fokkema IFAC , Kroon M, López Hernández JA et al. The LOVD3 platform: efficient genome-wide sharing of genetic variants. *Eur J Human Genet* 2021;29:1796–803. doi: 10.1038/s41431-021-00959-x 34521998 PMC8632977

[89] Sinha S , Black ML, Agarwal S et al. ThalInd, a β‐thalassemia and hemoglobinopathies database for India: defining a model country‐specific and disease‐centric bioinformatics resource. *Human Mutation* 2011;32:887–93. doi: 10.1002/humu.21510 21520336

[90] Coordinators NR . Database resources of the national center for biotechnology information. *Nucleic Acids Res* 2014;42:D7–17. doi: 10.1093/nar/gkt1146 24259429 PMC3965057

[91] Wang J , Kong L, Gao G et al. A brief introduction to web-based genome browsers. *Brief Bioinform* 2013;14:131–43. doi: 10.1093/bib/bbs029 22764121

[92] Vihinen M , Hancock JM, Maglott DR et al. Human variome project quality assessment criteria for variation databases. *Human Mutation* 2016;37:549–58. doi: 10.1002/humu.22976 26919176 PMC6317523

[93] Taschner P , Witsch-Baumgartner M. Minimal content requirements for genomic variant databases. Human Variome Project. 2018. https://www.humanvariomeproject.org/sdp/wg03-minimal-content-for-gene-variant-databases-lsdbs.html (12 May 2022, date last accessed).

[94] Den Dunnen JT , Dalgleish R, Maglott DR et al. HGVS recommendations for the description of sequence variants: 2016 update. *Human Mutation* 2016;37:564–69. doi: 10.1002/humu.22981 26931183

[95] Köhler S , Gargano M, Matentzoglu N et al. The human phenotype ontology in 2021. *Nucleic Acids Res* 2021;49:D1207–D1217. doi: 10.1093/nar/gkaa1043 33264411 PMC7778952

[96] Vasant D , Chanas L, Malone J et al. Ordo: an ontology connecting rare disease, epidemiology and genetic data. In: *Proceedings of ISMB*. Vol. 30, Boston 11-12 July 2014. researchgate. Net, 2014.

[97] Vasilevsky NA , Matentzoglu NA, Toro S et al. Mondo: unifying diseases for the world, by the world. *medRxiv* 2022. doi: 10.1101/2022.04.13.22273750

[98] François L , Eyll JV, Godard P. Dictionary of disease ontologies (DODO): a graph database to facilitate access and interaction with disease and phenotype ontologies. *F1000Research* 2020;9:942. doi: 10.12688/f1000research.25144.1

[99] Mitropoulou C , Webb AJ, Mitropoulos K et al. Locus‐specific database domain and data content analysis: evolution and content maturation toward clinical use. *Human Mutation* 2010;31:1109–16. doi: 10.1002/humu.21332 20672379

[100] Claustres M , Horaitis O, Vanevski M et al. Time for a unified system of mutation description and reporting: a review of locus-specific mutation databases. *Genome Res* 2002;12:680–88. doi: 10.1101/gr.217702 11997335

[101] O’Leary NA , Wright MW, Brister JR et al. Reference sequence (RefSeq) database at NCBI: current status, taxonomic expansion, and functional annotation. *Nucleic Acids Res* 2016;44:D733–45. doi: 10.1093/nar/gkv1189 26553804 PMC4702849

[102] NIHR Global Health Research Unit on Genomic Surveillance of AMR . Whole-genome sequencing as part of national and international surveillance programmes for antimicrobial resistance: a roadmap. *BMJ Glob Health* 2020;5:e002244. 10.1136/bmjgh-2019-002244 PMC7689591 33239336

[103] Vihinen M , den Dunnen JT, Dalgleish R et al. Guidelines for establishing locus specific databases. *Hum Mutat* 2012;33:298–305. doi: 10.1002/humu.21646 22052659

[104] Celli J , Dalgleish R, Vihinen M et al. Curating gene variant databases (LSDBs): toward a universal standard. *Hum Mutat* 2012;33:291–97. doi: 10.1002/humu.21626 21990126

[105] Ekong R , Vihinen M. Checklist for gene/disease-specific variation database curators to enable ethical data management. *Human Mutation* 2019;40:1634–40. doi: 10.1002/humu.23881 31347738

[106] Kountouris P , Stephanou C, Bento C et al. ITHANET: information and database community portal for haemoglobinopathies. *bioRxiv* 2017;1:209361. doi: 10.1101/209361

