# Supplementary material for: Global Globin Network and adopting genomic variant database requirements for thalassemia
Source: Database (Oxford). 2024 Sep 4;2024:baae080. doi: 10.1093/database/baae080 (PMC11373567; doi:10.1093/database/baae080)
Supplement: baae080_Supp [file baae080_supp.zip › suppl_data/Supplementary Materials.DOCX]

**Supplementary Materials**

**Terms and definitions**

1. **Variant data**: Variant data are data describing changes to the sequence of DNA, RNA, or protein, and their functional and/or clinical relevance, as well as any additional relevant annotations and information about these changes. It may be structured as variant-level data or case-level data.

- Variant-level data are a subset of genomic variant data in which the primary unit of data is the variant and its associated attributes. Variant-level data also provide reliable references/links to other databases (e.g., functional databases, variant frequency databases). In contrast to case-level data, sharing variant-level data normally does not require consent, because they are unlikely to uniquely identify a person.
- Case-level data are a type of genomic variant data in which the primary unit of data is a single individual's variant. Typical data include genotype (single variant to complete genomic dataset) and phenotype (e.g., diagnosis, presence or absence of phenotypic characteristics, family history) and may also include demographic information (e.g., ethnicity, population, age, sex, geographical origin). The database environment for sharing case-level data (e.g., public, registered access, controlled access) is often dependent on the sort of data being shared, its possibility of being personally identifiable, the type of consent, and local/national legislation.

1. **Variant classification**: Variant classification refers to the process of determining the clinical or functional relevance of a certain variant by evaluating all available data. Interpretations and claims of clinical significance may include whether the variant is deemed causative of a particular disease phenotype, whether it modulates drug response, confers sensitivity to drugs or other bioactive agents, protects against disease, or is associated with specific conditions as risk factors. Mendelian disease (1), somatic cancer (2), pharmacogenomics (3) and hereditary cancer (4) as the standards have been published to define terminologies and approaches to evaluate evidence, and corresponding classification criteria exclude polygenic disease and traits.
2. **Assertion**: An assertion in the context of genomic variant data is a claim about the functional and/or clinical significance of a specific variant. The process of variant classification results in assertions.

1 = has no known functional effect (“benign / not pathogenic”), Class 1;

3 = probably has no functional effect (“probably benign / not pathogenic”), Class 2;

5 = variant of unknown significance = VUS, Class 3;

7 = probably has a functional effect (“probably pathogenic”), Class 4;

9 = has a functional effect (“pathogenic”), Class 5;

NC = not classified

1. **Clinical significance**: Clinically relevant variants in genomic variation data include those that have been linked to a specific illness phenotype, regulate drug responses, confer sensitivity to drugs or other bioactive agents, provide protection against disease, or are linked to specific disorders as risk factors. Clinically actionable variations are those that represent a significant danger to the health of those who possess them. Medical intervention and preventative measures may be among the options. Supplementary Table 1 shows the list of phenotypic data (clinical data) that are informative for the clinical significance of thalassemic variants.

Supplementary Table S1. List of informative phenotypic data for thalassemia patients

| ID | Heading |
| --- | --- |
| Phenotype | Phenotype details |
| Genotype | Genotype details |
| Age/Thal | Age at thalassemia presentation |
| Age_first_blood_transfusion | Age at first blood transfusion |
| Frequency_blood_transfusion | Frequency of blood transfusion |
| Age_current_transfusion | Age at current transfusion |
| Blood_transfusion | Number of blood transfusion |
| Hematological_parameters   1. Hb (g/dL) 2. MCV (fL) 3. MCH (pg) 4. HbA2 (%) 5. HbF (%) 6. Other hemoglobin variants | Hematological parameters at diagnosis |
| Splenectomy_done | Splenectomy done |
| Size | Size of spleen (cm) |
| Current_serum_ferritin_level | Current serum ferritin level |
| Iron_chelation | Iron chelation |
| Age/weight | Age per weight |
| Height/weight | Height per weight |
| Height_velocity | Height velocity |
| Mid_parental_height | Mid parental height |

1. **Functional significance (or Functional consequence):** The effect of a change in the sequence of DNA or RNA on the resulting gene product is referred to as a variant's functional significance.
2. **Demographic data**: Demographic data at the variant and case levels may contain individual or aggregated information about age, gender, ethnicity, population and geographical origin.
3. **Attribute**: Attribute is a type of information that is linked to a bigger notion or entity and can be valued. Entities in these guidelines are either variations or persons, and their characteristics include position within the genome, phenotype, functional relevance, and age. Theoretically, a large number of defined attributes and their respective values may be necessary to adequately represent an entity.
4. **Acceptable value**: The values, types of values, or ranges of values that can be assigned to a certain characteristic in the system under consideration represent acceptable values. To avoid ambiguity, it is best if certain values are utilized consistently across the database.

**Table S2. Data fields for the centralised and for local Thalassemia database installations based on the LOVD platform in MyHVP Database for thalassaemia**

| **Field** | **Description (where necessary)** |
| --- | --- |
| Gene  Symbol  Gene  Chromosome  Band  Transcripts  Variants  Number of unique variants  Associated with diseases | Gene: *HBB/HBA1/HBA2* |
| Transcripts  Chromosome  Gene ID  Gene name  NCBI ID  NCBI Protein  Number of Variants | Variants on *HBB/HBA1/HBA2* |
| **Variants**  Variants on transcripts  Affect function  Location  Exon  DNA change (Cdna)  Published as  Position  RNA change  Protein | Variants on transcripts of *HBB/HBA1/HBA2* |
| **Genomic Variants**  Individual ID  Chromosome  Allele  Affects function (reported)  Affects function (concluded)  Type  DNA change (genomic)  Reference  Frequency | Genomics variants on *HBB/HBA1/HBA2* transcripts |
| **Variants screenings**  Template Variants (DNA/RNA/Protein)  Technique  Tissue  Gene screened.  Variants found | Techniques of screening of *HBB/HBA1/HBA2* |
| **Individuals**  Gender  Geographic origin  Ethnic origin  Population  Disease  Gene screened  Variants in genes  Variants | Patients of thalassaemia |
| **Phenotype data on individual**  Age of onset  Phenotype details  Inheritance  Age at thalassaemia presentation  Age at receiving first blood transfusion (year)  Age at current transfusion (year)  Frequency of blood transfusion  Hematological parameters at diagnosis: Hb level (g/dL)  Hematological parameters at diagnosis: HbA2 (%)  Hematological parameters at diagnosis: HbF (%)  Hematological parameters at diagnosis: MCH (pg)  Hematological parameters at diagnosis: MCV (fL)  Hematological parameters at diagnosis: other Hb variants  Spleen size (cm)  Height per age  Height velocity  Mid parental height  Weight per age  Current serum ferritin level  Iron chelation  Splenectomy done | Phenotype and clinical parameter of thalassaemia patients |
| **Disease**  Abbreviation  Name  OMIM ID  Number of phenotype  Associated with genes  Associated tissues  Disease features | Thalassaemia disease |
| **Screenings**  Template (DNA/RNA/Protein)  Technique  Tissue  Gene screened  Variants found | Laboratory methods used to test the patients |

**Membership of Global Globin Network (GGN)**

The list of full membership of Global Globin Network (GGN) (in alphabetical order):

1. Abdul Halim Fikri Bin Hashim

Malaysian Node of the Human Variome Project Secretariat, School of Medical Sciences, Universiti Sains Malaysia, Health Campus, 16150 Kubang Kerian, Kelantan, Malaysia

2. Ahzad Hadi Ahmad

Regenerative Medicine Cluster, Advanced Medical and Dental Institute, Universiti Sains Malaysia, 13200 Kepala Batas, Pulau Pinang, Malaysia

3. Amanda Krause

National Health Laboratory Service (NHLS) & The University of the Witwatersrand, Room 104, First Floor, Watkins Pitchford Building, NHLS Braamfontein, Cnr Hospital and De Korte St, Hillbrow, P O Box 1038, Jo-hannesburg, South Africa

4. Angelo Lores Brunetta

Thalassaemia International Federation, Nicosia, Cyprus

5. Antonio Piga

Department of Clinical and Biological Sciences, Turin University, Turin, Italy

6. Ariffin Nasir

Department of Paediatrics, School of Medical Sciences, Universiti Sains Malaysia, 16150 Kubang Kerian, Kelantan, Malaysia

7. Atif Amin Baig

Faculty of Medicine, Universiti Sultan Zainal Abidin, 20400, Kuala Terengganu, Terengganu, Malaysia

8. Bin Alwi Zilfalil

Human Genome Centre, School of Medical Sciences, Universiti Sains Malaysia, Health Campus, 16150 Kubang Kerian, Kelantan, Malaysia

9. Carsten Werner Lederer

Molecular Genetics Thalassaemia Department, The Cyprus Institute of Neurology & Genetics, 6 Iroon Avenue, 2371 Ayios Dometios, Nicosia, Cyprus | PO Box 23462, 1683, Nicosia, Cyprus

10. Catherine Lynn T. Silao

Institute of Human Genetics, National Institutes of Health; Department of Pediatrics, College of Medicine, University of the Philippines, Manila, Philippines

11. Celeste Bento

Department of Haematology, Centro Hospitalar e Universitário de Coimbra (CHUC), Coimbra, Portugal

12. Chean Sophal

Royal Phnom Penh Hospital, Cambodia

13. Chris Arnold

BioGrid Australia, Hodgson Associates. 4 Hodgson St Kew, Victoria Australia. 3101

14. Coralea Stephanou

Molecular Genetics Thalassaemia Department, The Cyprus Institute of Neurology & Genetics, 6 Iroon Avenue, 2371 Ayios Dometios, Nicosia, Cyprus | PO Box 23462, 1683, Nicosia, Cyprus

15. Diana Abdul Rashid

Department of Paediatrics, School of Medical Sciences, Universiti Sains Malaysia, 16150 Kubang Kerian, Kelantan, Malaysia

16. Domenico Coviello

Laboratorio di Genetica Umana, IRCCS Istituto Giannina Gaslini, Largo Gerolamo Gaslini 5, 16147 Genova, Italy

17. Doris Lau Sie Chong

Department of Paediatrics, Hospital Canselor Tuanku Muhriz UKM (HCTM), Jalan Yaacob Latif, Bandar Tun Razak, 56000 Cheras, Kuala Lumpur, Malaysia

18. Ezalia Esa

Haematology Unit, Cancer Research Centre, Institute for Medical Research, National Institutes of Health, No. 1, Jalan Setia Murni U13/52, Seksyen U13, Bandar Setia Alam, 40170 Shah Alam, Selangor Darul Ehsan, Malaysia

19. Ghada El-Kamah

Clinical Genetics Department, Human Genetics and Genome Research Institute, National Research Centre Cairo, Egypt

20. Hafiza Alauddin

Department of Pathology, Faculty of Medicine, Universiti Kebangsaan Malaysia, 56000 Cheras, Kuala Lumpur, Malaysia

21. Hafizur Rahman

International Centre for Diarrhoeal Disease Research, Bangladesh, Dhaka, Mohakhali, Bangladesh

22. Helen Robinson

Nossal Institute for Global Health, MDDHS, University of Melbourne, Melbourne Australia

23. Henri Wajcman

U 955 INSERM Unit, Henri Mondor Hospital, Creteil, France

24. Hishamshah Mohd Ibrahim

Deputy Director-General of Health (Research and Technical Support), Ministry of Health, Malaysia

25. Humayun Iqbal

Services Institute of Medical Sciences, Lahore

26. Intan Juliana Abd Hamid

Advanced Medical and Dental Institute, Universiti Sains Malaysia, Bertam, Malaysia

27. Ita Margaretha Nainggalan

Eijkman Institute for Molecular Biology, Jakarta

28. Jacques Elion

Medical School, Université Paris Diderot

29. Johan T den Dunnen

Leiden University Medical Center, Leiden, South Holland, the Netherlands

30. John Burn

Translational and Clinical Research Institute, International Centre for Life Times Square Newcastle upon Tyne, NE1 3BZ, United Kingdom

31. John Porter

University College London, University College London Hospitals, London, United Kingdom

32. Julia Hasler

Global Variome, Institute of Genetic Medicine, International Centre for Life, Central Parkway, Newcastle upon Tyne, NE1 3BZ, United Kingdom

33. Lantip Rujito

Department of Molecular Biology, Faculty of Medicine, Jenderal Soedirman University, Purwokerto, Central Java, Indonesia

34. Law Hai Yang

Department of Paediatrics, KK Women’s and Children’s Hospital, Singapore

35. Leon Tshilolo

Centre Hospitalier Monkole, Kinshasa, Democratic Republic of Congo

36. Loh CKhai

Department of Paediatrics, Universiti Kebangsaan Malaysia Medical Centre Kuala Lumpur, Malaysia

37. Lukusa Tshilobo Prosper

Department of Internal Medicine, University Hospital of Kinshasa, Faculty of Medicine, University of Kinshasa, Kinshasa, Democratic Republic of Congo

38. Marina Kleanthous

Molecular Genetics Thalassaemia Department, The Cyprus Institute of Neurology & Genetics, 6 Iroon Avenue, 2371 Ayios Dometios, Nicosia, Cyprus | PO Box 23462, 1683, Nicosia, Cyprus

39. Mas Rina Wati

Institute of Health Sciences, University Brunei Darussalam, Brunei Darussalam, Brunei

40. Ming Qi

Department of Cell Biology and Medical Genetics, School of Medicine, Zhejiang University, Hangzhou, China.

41. Mohd Ismail Bin Ibrahim

Department of Community Medicine, School of Medical Sciences, Universiti Sains Malaysia, Kelantan, Malaysia.

42. Narazah Yusoff

Advanced Medical and Dental Institute, Universiti Sains Malaysia, Bertam 13200 Kepala Batas, Pulau Pinang Malaysia

43. Narimah Awin

National Population and Family Development Board, Bangunan LPPKN, No. 12B, Jalan Raja Laut, 50350 Kuala Lumpur

44. Nasrin Moazami

Department of Biotechnology, Iranian Research Organization for Science and Technology (IROST), Tehran, Iran

45. Nguyen Hoang Nam

Department of Clinical Hematology, National Children’s Hospital, Hanoi, Vietnam

46. Nguyen Thi Mai Huong

Department of Hemato-Oncology, National Children’s Hospital, Hanoi, Vietnam

47. Nik Norliza Nik Hassan

School of Health Sciences, Universiti Sains Malaysia, Health Campus, 16150 Kubang Kerian, Kelantan, Malaysia

48. Norafiza Mohd Yassin

Haematology Unit, Cancer Research Centre, Institute for Medical Research, National Institutes of Health, No. 1, Jalan Setia Murni U13/52, Seksyen U13, Bandar Setia Alam, 40170 Shah Alam, Selangor Darul Ehsan, Malaysia

49. Norhaza Abdul Rahim

Hospital Pulau Pinang, Jalan Residensi 10990 Pulau Pinang, Malaysia

50. Norsarwany Mohamad

Department of Paediatrics, School of Medical Sciences, Universiti Sains Malaysia, 16150 Kubang Kerian, Kelantan, Malaysia

51. Norunaluwar Jalil

UKM Specialist Children’s Hospital, Jalan Yaacob Latif, Bandar Tun Razak, 56000 Cheras, Kuala Lumpur, Malaysia

52. Nur Aisyah Aziz

Institute for Medical Research, Jalan Pahang, 50588 Kuala Lumpur, Wilayah Persekutuan Kuala Lumpur

53. Nur Azuar Abdul Rahim

Advanced Medical and Dental Institute, Universiti Sains Malaysia, Bertam 13200 Kepala Batas, Pulau Pinang Malaysia

54. Nurul Fatihah Azman

Department of Paediatrics, School of Medical Sciences, Universiti Sains Malaysia, 16150 Kubang Kerian, Kelantan, Malaysia

55. Oyekami Nash

National Biotechnology Development Agency, Abuja, Nigeria 10099

56. Petros Kountouris

Molecular Genetics Thalassaemia Department, The Cyprus Institute of Neurology & Genetics, 6 Iroon Avenue, 2371 Ayios Dometios, Nicosia, Cyprus | PO Box 23462, 1683, Nicosia, Cyprus

57. Qasim Ayub

Monash University Malaysia, Subang Jaya, Selangor, Malaysia

58. Rabiatul Basria S.M.N. Mydin

Advanced Medical and Dental Institute, Universiti Sains Malaysia, Bertam 13200 Kepala Batas, Pulau Pinang Malaysia

59. Raj Ramesar

Department of Pathology, University of Cape Town City of Cape Town, Western Cape, South Africa

60. Raja Zahratul Azma Raja Sabudin

Department of Pathology, Faculty of Medicine, Universiti Kebangsaan Malaysia Medical Centre, Jalan Yaacob Latif, Bandar Tun Razak 56000 Cheras, Kuala Lumpur, Malaysia

61. Ramdan Panigoro

Department of Biomedical Sciences, Medical Genetics Research Center, Faculty of Medicine, Universitas Padjadjaran, Bandung, West Java Province, Indonesia

62. Reena Das

Department of Hematology, Postgraduate Institute of Medical Education and Research, Sector 12, Chandigarh 160012, India

63. Rosemary Ekong

Department of Genetics, Evolution and Environment, University College London, London, UK

64. Rosline Hassan

Department of Haematology, School of Medical Sciences, Universiti Sains Malaysia, 16150, Kubang Kerian, Kelantan, Malaysia

65. Rosnah Bahar

Department of Haematology, School of Medical Sciences, Universiti Sains Malaysia, 16150, Kubang Kerian, Kelantan, Malaysia

66. Saidatul Norbaya Buang

Family Health Development Division, Ministry of Health Malaysia, Wilayah Persekutuan Putrajaya, Malaysia

67. Sharifah Nany Rahayu Karmilla Syed Hassan

Malaysian Node of the Human Variome Project Secretariat, School of Medical Sciences, Universiti Sains Ma-laysia, Health Campus, 16150 Kubang Kerian, Kelantan, Malaysia

68. Siti Nor Assyuhada Mat Ghani

School of Health Sciences, Universiti Sains Malaysia, Health Campus, 16150 Kubang Kerian, Kelantan, Malaysia

69. Sri Mulatsih

Department of Child Health, Faculty of Medicine, Public Health and Nursing, Universitas Gadjah Mada/Dr Sardjito Hospital, Yogyakarta, Indonesia

70. Suria Emilia Suhana Othman

Advanced Medical and Dental Institute, Universiti Sains Malaysia, Bertam 13200 Kepala Batas, Pulau Pinang Malaysia

71. Suthat Fucharoen

Thalassemia Research Centre, Institute of Molecular Biosciences, Mahidol University, Thailand

72. Thong Meow Keong

Department of Paediatrics, Faculty of Medicine, Universiti Malaya, Kuala Lumpur

73. Tilak Shrestha

Molecular Biology Unit, Central Department of Biotechnology, Tribhuvan University, Kathmandu, Nepal

74. Veena Selvaratnam

Hospital Ampang, Jalan Mewah Utara, Taman Pandan Mewah, 68000 Ampang Jaya, Selangor, Malaysia

75. Vip Viprakasit

Department of Paediatrics and Thalassaemia Centre, Siriraj Hospital, Mahidol University, Thailand

76. Wardah Yusof

Malaysian Node of the Human Variome Project Secretariat, School of Medical Sciences, Universiti Sains Ma-laysia, Health Campus, 16150 Kubang Kerian, Kelantan, Malaysia

77. Yetty Hernaninsih

Faculty of Medicine, Universitas Airlangga, Indonesia.

78. Yetty Movieta Nency

Department of Paediatrics, Faculty of Medicine, Universitas Diponegoro/RS Dr Kariadi, Semarang, Indonesia

79. Zarina Abdul Latiff

Department of Paediatrics, Faculty of Medicine, Universiti Kebangsaan Malaysia Medical Centre, Cheras, 56000 Wilayah Persekutuan (Kuala Lumpur), Malaysia

80. Zulaiha Muda

Hospital Kuala Lumpur, Jalan Pahang Kuala Lumpur 50586 Wilayah Persekutuan (Kuala Lumpur) Malaysia

**References**

1. Richards S, Aziz N, Bale S, Bick D, Das S, Gastier-Foster J et al. Standards and guidelines for the interpretation of sequence variants: a joint consensus recommendation of the American College of Medical Genetics and Genomics and the Association for Molecular Pathology. *Genetics in medicine*. (2015) 17:405-23.
2. Li MM, Datto M, Duncavage EJ, Kulkarni S, Lindeman NI, Roy S et al. Standards and guidelines for the interpretation and reporting of sequence variants in cancer: a joint consensus recommendation of the Association for Molecular Pathology, American Society of Clinical Oncology, and College of American Pathologists. *The Journal of molecular diagnostics*. (2017) 19:4-23.
3. Caudle KE, Dunnenberger HM, Freimuth RR, Peterson JF, Burlison JD, Whirl-Carrillo M et al. Standardizing terms for clinical pharmacogenetic test results: consensus terms from the Clinical Pharmacogenetics Implementation Consortium (CPIC). *Genetics in Medicine*. (2017) 19:215-23.
4. Plon SE, Eccles DM, Easton D, Foulkes WD, Genuardi M, Greenblatt MS et al. IARC Unclassified Genetic Variants Working Group: Sequence variant classification and reporting: recommendations for improving the interpretation of cancer susceptibility genetic test results. *Human Mutation*. (2008) 29:1282-91.
